# GWAS Based on RNA-Seq SNPs and High-Throughput Phenotyping Combined with Climatic Data Highlights the Reservoir of Valuable Genetic Diversity in Regional Tomato Landraces

**DOI:** 10.3390/genes11111387

**Published:** 2020-11-23

**Authors:** Monica Rodriguez, Alessandro Scintu, Chiara M. Posadinu, Yimin Xu, Cuong V. Nguyen, Honghe Sun, Elena Bitocchi, Elisa Bellucci, Roberto Papa, Zhangjun Fei, James J. Giovannoni, Domenico Rau, Giovanna Attene

**Affiliations:** 1 Dipartimento di Agraria, Università degli Studi di Sassari, 07100 Sassari, Italy; alessandro.scintu@gmail.com (A.S.); cmposadinu@uniss.it (C.M.P.); dmrau@uniss.it (D.R.); attene@uniss.it (G.A.); 2 Centro per la Conservazione e Valorizzazione della Biodiversità Vegetale—CBV, Università degli Studi di Sassari, 07041 Alghero, Italy; 3 Boyce Thompson Institute for Plant Research and U.S. Department of Agriculture—Agriculture Research Service, Ithaca, New York, NY 14853, USA; yx25@cornell.edu (Y.X.); sunhonghe_1984@163.com (H.S.); zf25@cornell.edu (Z.F.); jjg33@cornell.edu (J.J.G.); 4 Global Institute for Food Security, University of Saskatchewan, Saskatoon, SK S7N 0W9, Canada; cuong.nguyen@gifs.ca; 5 Dipartimento di Scienze Agrarie, Alimentari e Ambientali—D3A, Università Politecnica delle Marche, 60131 Ancona, Italy; e.bitocchi@staff.univpm.it (E.B.); e.bellucci@staff.univpm.it (E.B.); r.papa@staff.univpm.it (R.P.)

**Keywords:** *Solanum lycopersicum* L., landraces, genomic diversity, digital phenotyping, RNA-Seq, genome-wide association study (GWAS)

## Abstract

Tomato (*Solanum lycopersicum* L.) is a widely used model plant species for dissecting out the genomic bases of complex traits to thus provide an optimal platform for modern “-omics” studies and genome-guided breeding. Genome-wide association studies (GWAS) have become a preferred approach for screening large diverse populations and many traits. Here, we present GWAS analysis of a collection of 115 landraces and 11 vintage and modern cultivars. A total of 26 conventional descriptors, 40 traits obtained by digital phenotyping, the fruit content of six carotenoids recorded at the early ripening (breaker) and red-ripe stages and 21 climate-related variables were analyzed in the context of genetic diversity monitored in the 126 accessions. The data obtained from thorough phenotyping and the SNP diversity revealed by sequencing of ripe fruit transcripts of 120 of the tomato accessions were jointly analyzed to determine which genomic regions are implicated in the expressed phenotypic variation. This study reveals that the use of fruit RNA-Seq SNP diversity is effective not only for identification of genomic regions that underlie variation in fruit traits, but also of variation related to additional plant traits and adaptive responses to climate variation. These results allowed validation of our approach because different marker-trait associations mapped on chromosomal regions where other candidate genes for the same traits were previously reported. In addition, previously uncharacterized chromosomal regions were targeted as potentially involved in the expression of variable phenotypes, thus demonstrating that our tomato collection is a precious reservoir of diversity and an excellent tool for gene discovery.

## 1. Introduction

Tomato (*Solanum lycopersicum* L.) is a major vegetable crop worldwide and a widely used model species in genomic studies and breeding. The rapidly increasing availability of genomic data has allowed researchers to bypass the limitations of a single reference genome and has facilitated development of a pan-genome that represents the genetic diversity of cultivated and wild tomato species [1]. Recent groundbreaking studies have shown how the integration of widely variable tomato collections and multi-omics tools can contribute to advances in population genomics, domestication and plant evolution studies [2,3,4].

Our interest in tomato derives from the relevance of this crop for the Mediterranean area, where Italy is the leading European tomato producer, with an overall production in 2019 of ~6 million tons and a harvested area of ~100,000 ha (FAOSTAT, 2018; ISTAT, 2018, 2019). In Sardinia in particular, together with artichoke, tomato is the most important horticultural crop species, with numerous landraces still cultivated locally by farmers. This is of special relevance if we consider that landraces might be directly used in marginal environments or exploited in future breeding programs as a reservoir of genes that underlie quality and productive traits that can be used to increase the performance of modern cultivars and to boost sustainable agriculture [5,6,7,8,9]. As noted by Lin and colleagues [2], modern tomato cultivars have arisen following years of domestication and breeding, which have progressively reduced the genomic diversity, together with the chances of improving the species through conventional breeding. Therefore, it is important that researchers work to preserve and expand the genetic diversity through the analysis of local and regional genotypes. The results of these studies will help in disentangling the role of different genes in the expression of important agronomic traits [2,10,11]. Transcriptomic data, in particular, have been used to both compare expression profiles (expression quantitative trait loci (eQTLs)) in plant populations at different development stages, and genotype (using RNA-Seq–derived single nucleotide polymorphisms (SNPs)) germplasm collections that can be used in genome-wide association studies (GWAS) and genomic selection analyses [10,12,13].

Genome-wide association studies have become a powerful tool to investigate and link the huge available amounts of genome sequence variation data with measurable phenotypic variations [2,3,4]. Recent examples in tomato can be found for conventional fruit and plant traits and metabolites [2,3,14,15,16]. Typically, GWAS is performed by using a single-locus mixed linear model (MLM) that incorporates both population structure and kinship to limit false genotype–phenotype associations [17]. Concurrent with the increased use of GWAS, continuous improvements are being implemented to overcome computational limits and additional pitfalls [17,18,19,20,21,22]. For example, multilocus mixed linear models (MMLMs) are considered more appropriate when complex traits are investigated, because these incorporate multiple markers simultaneously as covariates in a step-wise MLM [19]. An improved multilocus model, known as fixed and random model circulating probability unification (FarmCPU), can help to remove the confounding effects between markers incorporated into the model and kinship [21]. A further method that does not require population structure information was also designed, known as the quantitative trait cluster association test (QTCAT); when compared to standard GWAS, QTSAT revealed novel associations [22].

Here we used both conventional MLM and the two alternative models, FarmCPU and QTCAT, to scan an ad hoc assembled population of 120 tomato varieties that were mostly landraces for relevant associations among 2470 RNA-Seq–derived SNPs and traits of interest. In this “multi-omics” era, where DNA sequence data are easily acquired, phenotypic data are becoming the limiting factor. For this reason, we have here mainly addressed our efforts at thoroughly characterizing this population. Accordingly, the aim of the present study was to link the diversity observed at the phenotypic level with that observed at the transcribed genome level. To achieve this goal, we used three different GWAS approaches that provided an output of 536 significant associations related to plant and fruit morphophenological traits, fruit quality and response to climate. With the present study, we are acknowledging the value of this tomato collection for future gene-function, genome-editing and crop-breeding studies.

## 2. Materials and Methods

### 2.1. Plant Materials and Experimental Design

We investigated a collection of 126 cultivated tomato (*Solanum lycopersicum* L.) accessions that comprised 64 landraces from Sardinia, 8 landraces from other regions of Italy, 43 landraces from different countries and 11 vintage or modern cultivars (Supplementary Table S1). The Sardinian landraces were mainly collected during 2006 and 2007, after being cultivated locally for ≥30 years, according to the farmers [23]. Forty-three landraces were provided by the Centre for Genetic Resources (CGN), Wageningen University (Wageningen, The Netherlands) and 14 accessions were kindly provided by Prof. Andrea Mazzucato, University of Tuscia (Viterbo, Italy), as specified in Supplementary Table S1.

We characterized the accessions phenotypically across two experimental trials that were run in Sardinia (Italy) during 2012 and 2013. We conducted the open-field trial in 2012 at Oristano, according to a randomized complete block design with five replicates, 124 treatments (accessions) and four plants per plot. The field trial started in June and the full mature fruit were harvested in September. In 2013, we set up the second trial from January to July in a greenhouse in Ottava (Sassari); a randomized complete block design was again used, with three replicates, 126 treatments (accessions) and three plants per plot.

### 2.2. Phenotypic Analysis

We used 26 conventional morphophenological traits (Supplementary Table S2) to characterize the plants and fruit of each accession, with the recording of: Days to flowering from sowing date (DTFs, days), days to flowering from transplanting date (DTFt, days), flowering-ripening interval (FRI, days), plant growth type (PGT, score), number of flowers per inflorescence (NFI), inflorescence type (ITP, score), stigma exertion (SE, score), leaf attitude (LAT, score), leaf length (LLE, cm), leaf width (LWI, cm), leaf length-to-width ratio (LL/W), foliage density (FD, score), fruit weight (FWG, g), fruit length (FLE, cm), fruit width (FWI, cm), fruit length-to-width ratio (FL/W), fruit color (FCO, score), fruit shape (FSH, score), green shoulder (GRS, score), pistil scar shape (PSS, score), blossom end shape (BES, score), cross-sectional shape (CSS, score), number of locules (NOL), puffiness appearance (PUF, score), pericarp thickness (PTK, mm) and degrees Brix (BRIX, Bx). These descriptors were chosen from the guidelines of Bioversity International (http://tinyurl.com/n7k75m6).

In addition to these conventional descriptors, we carried out digital phenotyping on the mature fruit collected in 2013 using the Tomato Analyzer software [24,25]. For each accession, we analyzed three longitudinal and three transversal sections for a total of six fruit. The 300 dpi JPEG images were acquired with a scanner (Mustek Must A3 600S, Mustek Europe B.V., Oosterhout, The Netherlands) and analyzed morphometrically with the Tomato Analyzer v. 3 software [24,25,26]. We chose 44 traits (Supplementary Table S2) from the attribute list, 35 from the longitudinal section, representing the following main descriptors: Seven basic measurements (Perimeter, Area, Width Mid-height, Maximum Width, Height Mid-width, Maximum Height, Curved Height); three fruit shape indices (Fruit Shape Index External I, Fruit Shape Index External II, Curved Fruit Shape Index); three blockiness measures (Proximal Fruit Blockiness, Distal Fruit Blockiness, Fruit Shape Triangle); three homogeneity measures (Ellipsoid, Circular, Rectangular); four proximal fruit end shape indices (Shoulder Height, Proximal Angle Micro, Proximal Angle Macro, Proximal Indentation Area); four distal fruit end shape indices (Distal Angle Micro, Distal Angle Macro, Distal Indentation Area, Distal End Protrusion); six asymmetry measures (Obovoid, Ovoid, V. Asymmetry, H. Asymmetry Obovoid, H. Asymmetry ovoid, Width Widest Position); and five internal eccentricity measures (Eccentricity, Proximal Eccentricity, Distal Eccentricity, Fruit Shape Index Internal, Eccentricity Area Index). Nine traits were selected from the transversal section: Six basic measurements (Perimeter, Area, Width Mid-height, Maximum Width, Height Mid-width, Maximum Height); and three traits specific for the transversal section (Lobedness Degree, Pericarp Area, Pericarp Thickness). The settings were: Centimeters as units; for blockiness, position 0.9 was used as the upper position and 0.1 as the lower position, unless otherwise requested in a few specific cases; for proximal and distal angles, 20 degrees was used as the macro distance and three degrees as the micro distance (unless otherwise requested in a few specific cases).

### 2.3. Carotenoids

We extracted the carotenoids from three replicates of each accession (one per block), at both the breaker stage (i.e., early ripening), when the fruit are still rich in chloroplasts and the red-ripe stage, when the fruit accumulate large quantities of carotenoids, for a total of 762 samples. All of the harvested fruit at the breaker and red-ripe stages were healthy and uniformly colored within their stage. An example of the two ripening stages is shown in Supplementary Figure S1. At ripe stage, accessions with mature orange, yellow or pink fruit were harvested when the color reached maximum intensity. The harvested fruit were left for 6 h on a laboratory bench at room temperature to reduce harvest stress and ethylene production. Then they were cut into halves and separated from the seeds, frozen in liquid nitrogen and stored at −80 °C.

Frozen flesh samples from each fruit stage were rapidly homogenized and carotenoids extraction performed as described in [27]. Carotenoid detection was performed on a Summit HPLC system with a photodiode array detector (PDA-100; Dionex, Sunnyvale, CA, USA). Spectra were collected at 286 nm, 450 nm and 471 nm and pigments were identified via co-migration with purified standards and/or by their pigment-specific absorbance spectra. The list of carotenoids analyzed for each stage is shown in Supplementary Table S2.

### 2.4. Climatic Data

For each accession for which geographic coordinates were available (108 accessions), climatic data were also downloaded relative to each collection site. Bioclimatic variables were used for further comparative analyses with phenotypic and molecular data, as they are considered more biologically meaningful than monthly temperatures and rainfall. Indeed, they describe annual temperature and precipitation trends, seasonality or extreme environmental factors (e.g., temperature of the coldest and warmest month or precipitation of the wettest and driest quarters). The DIVA-GIS 7.5 software (http://www.diva-gis.org/) was used to extract ecological data from the free-access database at http://www.diva-gis.org/climate.

### 2.5. Transcriptome Sequencing and SNP Calling

Transcriptome sequencing was performed on 120 of the 126 accessions cultivated during the green-house trial in 2013. The fruits used for RNA extraction were the same used for carotenoid extraction (see above) Total RNA was extracted using RNeasy Plant Mini kits (Qiagen, Hilden, Germany) from full-ripe tissue samples previously frozen at −80 °C and homogenized. Strand-specific RNA-Seq libraries were constructed using a protocol described in [28] and 51-bp-long single-end reads were sequenced using a HiSeq 2000 platform (Illumina, San Diego, CA, USA). Raw RNA-Seq reads were processed to trim low-quality and adapter sequences using Trimmomatic [29]. The resulting cleaned RNA-Seq reads were aligned to the “Heinz” reference genome (version SL2.40) using STAR [30]. Duplicated reads in each RNA-Seq library were marked using Picard (http://broadinstitute.github.io/picard/) and only uniquely mapped reads were kept. SNPs were then identified based on the mpileup files generated by SAMtools [31]. The resulting raw SNPs (73,859) were filtered to exclude those with missing rate >0.9 and minor allele frequency <0.03, which obtained a final dataset of 2470 SNPs for downstream population genomic and GWAS analyses.

### 2.6. Statistical and Population Genomic Analyses

Ranges and mean values were calculated by site for each accession on quantitative conventional descriptors, Tomato Analyzer descriptors and carotenoid contents. Analysis of variance (ANOVA) was then performed on the conventional descriptors collected from the 122 accessions shared among the two sites. To test for significant variations among environments and genotypes and to estimate the size of the genotype by environment interaction (GxE) we used the model given in Equation (1):

Y_ijr_ = μ + G_i_ +E_j_ + G*E_ij_ + B_r_(E_j_) + ε_ijr_ (1)

where Y_ijr_ is the observation of the ith genotype, in the jth environment and block replicate r, μ is the grand mean, G_i_ is the effect of the ith genotype, E_j_ is the effect of the jth environment, G*E_ij_ is the interaction of the ith genotype with the jth environment, B_r_(E_j_) is the effect of the rth replicate in the jth year and ε_ijr_ is the random error.

The adjusted means (as the best linear unbiased predictors (BLUPs)) and the broad sense heritability were also calculated across sites for the quantitative conventional descriptors and within sites for the Tomato analyzer descriptors and the carotenoid contents. To calculate these statistics, we have fitted a mixed linear model based on the restricted maximum likelihood method with years as fixed effects and genotypes and blocks as random effects using Equation (2):

Yijr = μ + Gi +Ej + Br(Ej) + εijr(2)

where Yijr is the observation of the ith genotype, in the jth environment and block replicate r, μ is the grand mean, Gi is the effect of the ith genotype, Ej is the effect of the jth environment, G*Eij is the interaction of the ith genotype with the jth environment, Br(Ej) is the effect of the rth replicate nested in the jth year and εijk is the random error. 

The expression given in Equation (3) was used to calculate the statistics:

H^2^_B_ = σ^2^_g_/σ^2^_p_ (3)

where σ^2^_p_ = σ^2^_g_ + (σ^2^_g_⋅_e/_n_e_) + (σ^2^_ϵ__/_n_e_⋅n_r_) for heritability across the two sites and σ^2^_p_ = σ^2^_g_ + (σ^2^_ϵ__/_n_e_⋅n_r_) for heritability calculated by site, and where σ^2^_g_ is the genotypic variance, σ^2^_p_ is the phenotypic variance, σ^2^_g_⋅_e_ is the genotype by environment interaction, n_e_ is the number of environments, σ^2^_ϵ_ is the residual variance and n_r_ is the number of replicates. 

All the above statistical analyses were carried out using R dedicated packages (*lme4* and *car*) [32].

The Shannon–Weaver index was used to evaluate the diversity among the qualitative traits [33]. GenAlEx 6.5 [34] was used to calculate the formula H’ = $-\overset{n}{\underset{i=1}{\sum }}pilogb$, where *pi* is the frequency proportion of the descriptor state and *b* is the base of the logarithm. Most studies use natural logarithms, although some use base 2 (which makes no significant differences). Each value was normalized by dividing it by its maximum value (log2n, where n is the number of states) to keep the values between 0 and 1. Pearson’s correlations were estimated among the different traits using the *cor* standard R function and plotted using the *corrplot* package [35]. Principal component analysis (PCA) and cluster analysis based on all of the morphophenological and quality traits were also performed. The PCA results were plotted using a modified version of the *ggbiplot* package (https://github.com/vqv/ggbiplot). The Hopkins statistic [36] was used to assess the clustering tendency and the most likely number of groups was detected using the NbClust R package [37]. The Euclidean distance and the Ward method were used to draw the clusters and the results were plotted using the *factoextra* R package (https://cran.r-project.org/web/packages/factoextra/index.html).

Genetic diversity analyses were performed using GenAlEx 6.5 [34] and Arlequin vs. 3.5.1.2 [38], to calculate the main diversity statistics: Number of observed (Na) and expected (Ne) alleles, number of private alleles (PAs), expected heterozygosity (He) and unbiased expected heterozygosity (uHe). Hierarchical analysis of molecular variance (AMOVA) was performed to evaluate the partitioning of the genetic variance into, among and within groups of accessions, defined as: Sardinian landraces (SLRs), exotic landraces (ELRs) and modern or vintage cultivars (CVs). Genetic distances among groups were also calculated by pairwise F_ST_ values and also proportion of shared alleles between pairs of sub-populations.

A Mantel correlation test was then used to compare the genetic and phenotypic distances using the *mantel.rtest* function of the *ade4* R package; 9999 permutations were used to evaluate the significance of the test. The phenotypic distances were those obtained to draw the clustering plot, while the genetic Nei minimum distances were obtained using the R *adegenet* [39] and *popr* packages (URLhttps://grunwaldlab.github.io/poppr). 

To investigate population structure, we used the model-based clustering method, as implemented in Structure vs. 2.3.4 [40], and discriminant analysis of principal components (DAPC), implemented in the *adegenet* package for the R software [39]. The first method is a model-based approach that assigns each individual to different groups according to a membership coefficient (qi). We used an admixture model with the options “correlated allele frequencies among populations” and “infer the degree of admixture (a) by the data”. For each K (number of hypothetical populations), 20 runs (burn-in length, 100,000; iterations, 200,000) were carried out and the most likely number of K was determined using the method from Evanno et al. (2005), as implemented in the online program STRUCTURE Harvester [41]. The second method does not require any a priori knowledge of the population genetics model and among other advantages, it requires low computational effort to analyze large datasets and provides reliable assignment of individuals to groups [39]. The method uses PCA to transform the data and performs discriminant analysis on the principal components retained, thus also allowing easy graphical representation of the relatedness between the inferred groups. To run these analyses, we filtered the dataset of 2470 SNPs by clumping, to maintain only those with reduced pairwise linkage disequilibrium (r^2^ < 0.2). To filter the dataset, we used the R package *bigsnpr* [42], to obtain a dataset of 649 unlinked SNPs.

To further evaluate the structure of the population, we estimated the familial relatedness and linkage disequilibrium (LD) levels of the tomato collection by calculating the pairwise kinship coefficients as obtained from TASSEL 5.2.42 [43]. We then determined the intrachromosomal LD using both uncorrected estimations and estimates corrected for population structure and familial relatedness. In the first case, the classical r^2^ measure was calculated; in the second case, the r^2^ unbiased estimates were corrected by the structure of the sample (r_s_^2^), the relatedness of the individuals (r_v_^2^) or both (r_vs_^2^). These methods were implemented in the *LDcorSV* R package [44]. Pairwise distance between loci was calculated using *LD-vignette* included in the Bioconductor package *snpStats* implemented in R [45,46]. We then plotted the LD r^2^ data against the genetic distance and fitted the LD decay line as in [47], adapting an R script to our data (https://fabiomarroni.wordpress.com/). The regression line is based on [48] and the parameter C was calculated by using *SneP*, a program designed to easily estimate effective population size from genome-wide SNP data or directly from LD levels [49].

To identify the critical value of LD (r^2^ value) across our tomato population above which loci can be assumed to be associated, we used the method from [50]. Here, the LD threshold below which markers can be defined as unlinked and is defined based on the 95th percentile of a normalized distribution of markers located on different chromosomes. The intersection point between the regression line and the LD significance threshold was used to indicate the LD decay overall and along each chromosome.

### 2.7. Genome-Wide Association Studies

Genome-wide association studies were performed on the BLUPs of all of the collected traits by fitting a MLM implemented in GAPIT v.3 [51], which accounts for kinship (genotype relatedness) and population structure. To explore farther marker-trait associations, we used FarmCPU, also implemented in GAPIT v.3 [21,51]. FarmCPU is a modified multilocus mixed model approach that enhances the false-discovery rate and the QTL detection power by incorporating one or several markers as cofactors in a stepwise MLM, thus removing the confounding between testing markers and kinship; see e.g., [19]. Liu et al. [21] divided the modified multilocus mixed model approach into two parts: A fixed effect model and a random effect model, using these iteratively. To control for false positives, the fixed effect model tests markers one at a time and uses multiple associated markers as covariates. To limit an overcorrection of the model, the associated markers are then estimated in a random effect model and used to define kinship. To perform MLM and FarmCPU analyses, no missing data are allowed and we therefore imputed the missing phenotypic data using the *phenix* package implemented in R [52]. We also performed a relatively novel GWA analysis using QTCAT [22], a method that does not require population structure information to detect associations between markers and traits. This method, has been designed to overcome the limitations of single-locus MLM models that while correcting for population structure and genotype relatedness, cannot entirely avoid yielding spurious associations between a marker and a phenotype [22]. In particular, this QTCAT groups markers into clusters of correlated markers while simultaneously associating them to the phenotype and therefore it does not require any correction for the population structure.

## 3. Results

### 3.1. Phenotypic Traits Analyses

The ANOVA performed on the conventional quantitative phenotypic traits using year (*Y*), genotype (*G*) and genotype × year (*G × Y*) interactions as effects of the model revealed significant differences among the genotypes between the years, except for FWG, NFI and LWI (Table 1); significant strong variance (*p* < 0.001) was also observed for *G* and *G × Y* interaction effects (Table 1). The broad sense heritability (h^2^_B_) calculated across sites and by site also varied among conventional traits (Table 1). In particular, h^2^_B_ varied between 11.8% for FRI and 85.8% for FL/W. When calculated within the year, h^2^_B_ varied among the traits, with higher values were generally observed in 2013 than in 2012. Nonetheless, on average, h^2^_B_ was stable between the years, with mean h^2^_B_ values of 57.3% in 2012 and 59.5% in 2013. Wide and significant (*p* < 0.001) differences were observed in both 2012 and 2013 when looking at the differences among the genotypes, as shown by the mean, maximum and minimum values of the conventional quantitative traits (Supplementary Table S3).

Substantial variation was detected also for the conventional qualitative traits, as indicated by the Shannon–Weaver index (H’) (Supplementary Table S4). Almost all of the traits showed the same number of variants across the 2 years, with some relevant changes seen, e.g., for green stripes, with H’ of 0.50 in 2012 that was reduced to nearly zero in 2013 (greenhouse experiment). A similar trend was also observed for leaf attitude (H’__2012_ = 0.39, H’__2013_ = 0.13; Supplementary Table S4).

Significant differences among genotypes were also detected for the 44 Tomato Analyzer descriptors (Supplementary Table S5). Broad sense heritability was on average higher for these traits, with the highest value for pericarp thickness (h^2^_B_ = 92.21%) and the lowest for the perimeter of the longitudinal section (h^2^_B_ = 36.65%) (Supplementary Table S5). The mean value for all of the traits was ~70%.

For the carotenoids, the total content was higher at the ripe stage than the breaker stage, as expected (Supplementary Table S6). At the breaker stage, the total mean content of carotenoids was 6.09 μg/g fresh weight (fw) and as a general trend there was an increase of about 20-fold with full ripeness (121.55 μg/g fw). Moreover, the ANOVA output showed that all of the analyzed compounds showed significant differences among genotypes at both the breaker stage and the red ripe stages (Supplementary Table S6).

The heritability of the carotenoids was on average lower than the other phenotypic traits, with β-carotene showing the highest values at both breaker and ripe stages (45%, 57%, respectively) (Supplementary Table S6).

Both positive and negative correlations were found among the different traits (Supplementary Figure S2), with the most significant values observed between fruit size and shape (as recorded by both conventional and digital phenotyping). As an example, fruit weight (FWG) and locule number (NOL) were strongly and positively associated (r > 0.8, *p* < 0.0001) with the area, perimeter and maximum width (Width_M_H_L and Width_M_H_T) of the transverse and longitudinal sections of the fruit (as registered by Tomato Analyzer). Significant correlations were also observed between carotenoid content and fruit color and size. In particular, β-carotene content was negatively correlated (r = −0.4, *p* < 0.001) to the mean fruit weight (FWG) at both the breaker stage and the ripe stage and it was positively correlated with the degrees Brix at the breaker stage (r = 0.43, *p* < 0.0001) and the ripe stage (r = 0.20, *p* = 0.03). Fewer correlations were detected between bioclimatic variables and the plant/fruit traits, some of which were of particular note, such as those between the traits that described fruit size (e.g., FLE, FWG, FWI, area, perimeter, Max_W_L, Max_H_L, Max_W_T, Max_H_T) and bio3 (Isothermality), bio8 (Mean Temperature of Wettest Quarter), bio12 (Annual Precipitation), bio13 (Precipitation of Wettest Month), bio 16 (Precipitation of Wettest Quarter) and bio18 (Precipitation of Warmest Quarter). The observed correlations varied from −0.45 (*p* < 0.001) for correlations between FLE and bio3 and bio8, to −0.27 (*p* < 0.005) for correlations between area and perimeter with bio12, bio13 and bio16 (Supplementary Figure S2).

**Figure 1:**
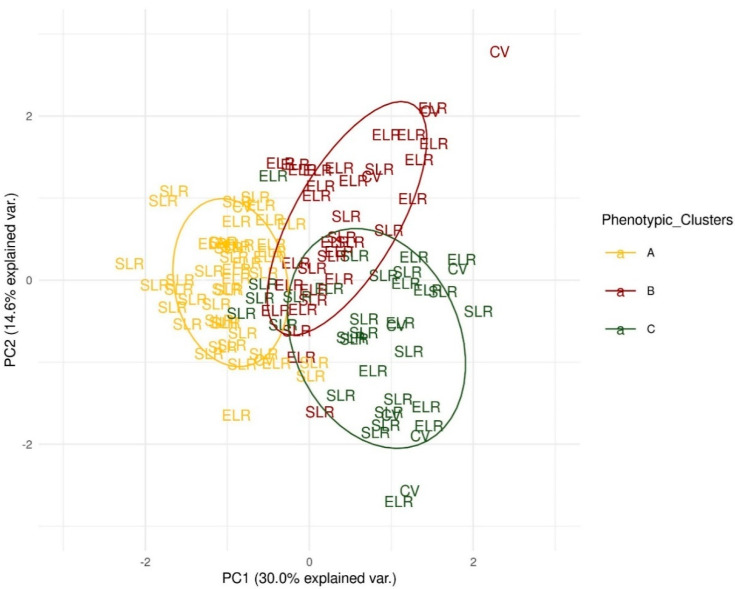
Principal component analysis performed on all of the morphophenological and fruit quality traits. ELR, exotic landraces; SLR, Sardinian landraces; CV, modern and vintage cultivars. Accessions are attributed to three phenotypic groups (yellow, green, red), based on the hierarchical clustering obtained with the same data.

Overall, a wide pattern of phenotypic variation was observed for all the traits, as shown by the PCA analysis, where the accessions were differentiated into three groups based on the hierarchical clustering obtained with the same data (Figure 1, yellow, red, green). The landraces from Sardinia were present in all of the three groups, as well as the exotic landraces and modern/vintage cultivars, thus showing that the subdivision into varietal types (i.e., ELR, SLR, CV) does not overlap with the clustering based on phenotypic variance. While we should remark that less than the 50% of the phenotypic variance is explained by the first two principal components.

The main features of the three groups can be deduced from the loading of the different traits on the first two principal components (Supplementary Table S7). Briefly, we can summarize that cluster A is constituted by plants with composite inflorescence and large fruit, with heterogeneous shapes, including both flattened and heart-shaped types, and usually characterized by a medium to thin pericarp and low BRIX degrees, with a higher *cis*-lycopene content. Cluster B mainly includes varieties of small size, mainly round shaped with a medium pericarp, high sugar content, high β-carotene (at both breaker and ripe stages) and low puffiness. Cluster C is mainly comprised of varieties of small/medium size, elongated fruit, with thick pericarp and intermediate phenotypes for sugar content.

### 3.2. Genetic Diversity and Structure

Table 2 gives the summary of the genetic diversity indices that indicate overall gene diversity (uHe) of 0.18, gene diversity that is the same for the Sardinian and exotic landraces (uHe = 0.16) and higher gene diversity within the group of cultivars (uHe = 0.29).

The genetic differentiation (F_ST_) among the groups was 0.07 on average and was always statistically significant. Moreover, the proportion of shared alleles indicated higher similarity between SLR and ELR than between CV and ELR (Table 3). On the other hand, SLR and ELR showed 11 and 50 private alleles, respectively, while only one private allele was detected in CV (Supplementary Table S8).

The partitioning of the SNP variance (AMOVA) indicated that only 4% of the total variation was due to a statistically significant differentiation (*p* < 0.001) among populations. This thus indicates that more than 96% of this diversity is due to the genetic variation among accessions within groups, either from different countries of the world or from a single country or region (e.g., Sardinia).

**Figure 2:**
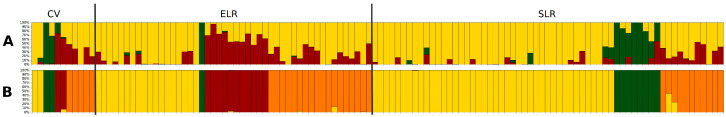
Population structure analysis as obtained from STRUCTURE (**A**) and DAPC (**B**) methods. (**A**) Accessions attributed to three genetic groups (yellow, green, red). (**B**) Accessions attributed to four genetic groups (yellow, green, orange, red). In both cases shown, each vertical bar indicates a single accession, which is colored according to the genetic group to which it was assigned. Accessions not assigned to a single group are colored according to the estimated proportion of membership to each genetic group and are defined as admixed. ELR, exotic landraces; SLR, Sardinian landraces; CV, modern and vintage cultivars.

Based on the Bayesian approach, the most likely number of genetic groups within this tomato population was three (Supplementary Figure S3a), while the DAPC method indicated four genetic groups (Supplementary Figure S3b). Looking in detail at these results, we found significant association between the methods in the assignment of the different accessions to the different genetic groups (Χ^2^ = 194.0, *p* < 0.0001). Indeed, if we do not consider the admixed individuals, the same accessions were attributed to the green and red genetic groups shown in Figure 2 by both methods, while the accessions attributed to the yellow group by STRUCTURE were split into two groups by DAPC (Figure 2, yellow, orange).

When looking at the composition of each genetic group, landraces from ELR and accessions from CV were assigned to all the three groups detected by STRUCTURE and to the four groups detected by DAPC. The main difference among the three varietal groups was seen for SLR, which showed rare occurrence of the red genetic group in Figure 2. This is evident at both K3 (STRUCTURE), where we observed a low level of membership of the SLR accessions to the red group and at K4 (DAPC), where there are no red bars. We further investigated whether there was any correlation between the genetic groups and the phenotypic clusters and despite the significant relationships between the genetic and phenotypic distances (Mantel test correlation = 0.38, *p* = 2 × 10^−4^; 9999 permutations), the genetic and phenotypic clustering do not completely overlap (Supplementary Figure S4).

### 3.3. Linkage Disequilibrium

Linkage disequilibrium that was calculated using the usual pairwise correlation measure (r^2^) was higher than the r^2^ measure corrected for population structure (r_s_^2^), kinship (r_v_^2^) and both population structure and kinship (r_vs_^2^). These were seen as r^2^ > r_s_^2^ > r_v_^2^ > r_vs_^2^; r_v_^2^ and r_vs_^2^ LD were very similar overall and within each chromosome (Table 4, Supplementary Figures S5 and S6).

**Figure 3:**
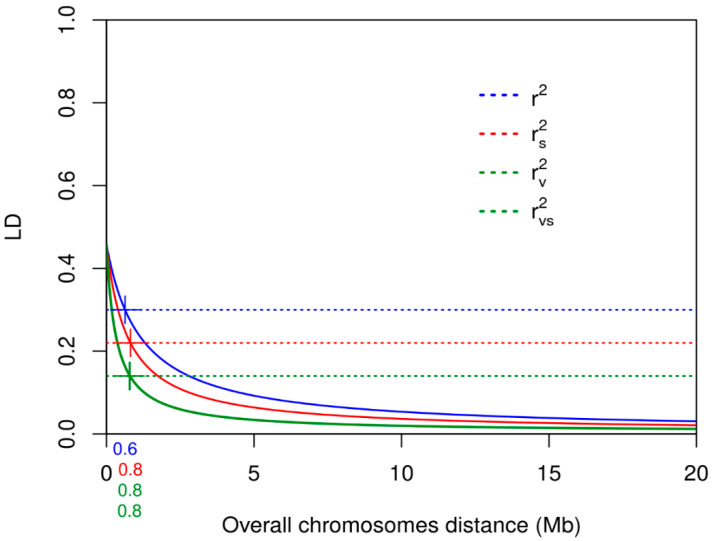
Linkage disequilibrium decay levels over all chromosomes calculated using the standard r^2^ coefficient and the r^2^ measure corrected for population structure (r_s_^2^), kinship (r_v_^2^) and both population structure and kinship (r_vs_^2^).

We plotted the r^2^, r_s_^2^, r_v_^2^ and r_vs_^2^ values against the genomic distances and modeled the LD decay for each chromosome (Figure 3) and the LD decay was determined at the intersection point between the regression curves and the LD thresholds. While the mean values of LD were lower for kinship corrected values, LD decayed at slightly higher distances for r_s_^2^ (1.40 Mb) and r_v_^2^ (1.37 Mb) than for standard r^2^ (1.06 Mb). This output was more evident within chromosomes for the r_s_^2^ decay.

Some differences were also observed for LD decay among the different chromosomes, with lower LD decay distances for chromosomes 1, 3, 8 and 10, while the highest decay distances were for chromosome 5 (Table 4.

### 3.4. Genome-Wide Association Studies

For clarity, we present the GWAS results according to trait investigated, as climate variables, fruit quality and fruit shape and size detected by conventional phenotyping and digital phenotyping, plant growth, inflorescence, leaf traits and phenology (Table 5; Supplementary Table S9). To interpret the GWAS results, we used chromosomal LD to determine the distance beyond which LD decay risked the possibility of spurious associations.

Among all the investigated traits, 536 marker trait associations (MTAs) were detected within 203 genes, 23 of which are genes (or homologs to *Arabidopsis* genes) that encode transcription factors (Table 5; Supplementary Tables S9 and S10). Among these 536 MTAs, 265 were detected by FarmCPU, 40 by GAPIT_MLM and 231 by QTCAT. Here, different SNPs were simultaneously detected by more than one method (Supplementary Table S9). 

### 3.5. Fruit and Plant Traits

In particular, the highest number of MTAs (388) was detected for fruit size and shape (derived from both conventional and digital phenotyping; Table 5). Among the 165 MTAs detected for tomato fruit size, 86 indicated a subset of 13 genes that were shared between the two sets of traits. Among the 223 MTAs detected for fruit shape by both phenotyping methods, 72 where located within 12 genes. 

Among the interesting MTAs we identified by both QTCAT and FarmCPU, one SNP (ch1_22) is located within the gene *Solyc01g010440*, which in addition to leaf size, was associated to multiple fruit traits (mainly related to fruit size) (Supplementary Tables S9 and S10). This gene was located 0.3 Mb from the *CRABS-CLAW* gene that encodes a YABBY transcription factor. On chromosome 2, one SNP was particularly relevant for fruit shape (ch2_151), which was detected by the three GWA models as associated to several traits (e.g., fruit shape, fruit size, BRIX, shape of pistil scar, shape of transverse section) and was located within the gene *Solyc02g081700*, which encodes a proteasome subunit α type protein, 13 kb from an *ANANTHA* gene (Supplementary Tables S9 and S10).

Among the genomic regions that influenced variation of fruit traits, an MTA on chromosome 4 (ch4_31) was 28 kb from a TONNEAU1 *Recruiting Motif* (*TRM*) gene that has been previously shown to influence final fruit shape [53].

On chromosomes 6 to 10, we detected more relevant MTAs for fruit size and shape, of which six were located within genes that encode transcription factors and others were either located near genes that influence fruit setting (OVATE-like, WUSCHEL-related, YABBY-like) or in regions where no genes determinant for the investigated fruit traits were found (Supplementary Table S9). On chromosome 11, there were other regions associated to fruit size and shape, among which the most significant was detected around 52 Mb, where the *FASCIATED* gene is located. *FASCIATED* has been extensively studied in tomato, where mutations to this gene result in altered floral meristem size and locule number [54,55,56,57,58].

Among the associations detected for quality traits, we found a relevant region on chromosome 1 (at 71 Mb) where three SNPs (ch1_38-40) were associated to fruit color and β-carotene content (Supplementary Table S9). These SNPs are located at 165 kb from *Solyc01g079620*, a *SlMYB12* gene that influences accumulation of the yellow-colored flavonoid (naringenin chalcone) in tomato fruit [59]. These MTAs were detected by MLM and FarmCPU.

Other relevant MTAs were detected for sugar content and other fruit quality traits. Among these, one is located within the gene *Solyc02g081700*, above mentioned for its association with different fruit size and shape traits and one located within *Solyc06g066320*, the gene encoding for a transcription factor homolog of Arabidopsis thaliana IWS1 (INTERACTS WITH SPT6) [60]. This gene was also associated to other traits related to fruit size and shape and inflorescence.

Marker-trait associations that correlated with other plant traits were also detected, such as growth habit, leaf traits and inflorescence traits. For plant growth, we found one SNP on chromosome 1 (ch1_153) within *Solyc01g108020*, a gene that encodes a thioredoxin M3, and one on chromosome 10 within *Solyc10g084400*, which encodes a glutathione S-transferase (Supplementary Table S9). These MTAs were located at 206 kb and 27 kb, respectively, from genes that encode an AP2-like ethylene-responsive transcription factor (*AP2/ERF*). Moreover, on chromosome 11 we detected a SNP (ch11_49) within a *DELLA* gene (*Solyc11g011260*) that encodes a gibberellic acid insensitive (GAI) transcription factor (Supplementary Table S9). The gene product belongs to a family that has been shown to have a role as redox regulator in hormone signaling pathways of different plant species, including *Arabidopsis,* rice and tomato [61,62,63,64,65].

Marker-trait associations for leaf size were also identified that included an MTA for leaf length within *Solyc04g080730*, at 31 kb from *Solyc04g080780* and 35 kb from *Solyc04g080790*, two BEL-like homeodomain genes [66]. BEL-LIKE genes have been shown to influence leaf development and morphology in many plant species, such as *Arabidopsis* and tomato [66,67,68]. More significant associations were found for flowering time (ch1_22, chr2_155, chr3_33), as well as for inflorescent type and number. In particular, looking at chromosome 11, two SNPs were located within *Solyc11g010490* at 82 kb from the J-1 gene, a MADS-box 512 transcription factor implicated in the differentiation of the pedicel abscission zone and the 513 maintenance of the inflorescence meristem [69,70] and a further one within *Solyc11g071680*, which encodes a serine/threonine kinase (homolog of the *Arabidopsis* TOUSLED). This is particular interesting because it encodes a serine/threonine protein kinase that is required for leaf and flower development and is involved in the regulation of RNA interference [71,72]. These functions are congruent with the other marker-trait associations that we have detected for this gene (i.e., fruit size and shape).

### 3.6. Climatic Data

Genome-wide association analysis on bio-climatic data relative to each collection site resulted in numerous MTAs distributed among all of the chromosomes (Table 5). Among these MTAs detected, one on chromosome 2 (ch2_130) is located within *Solyc02g071510*, a transcription factor GTE12, which is associated with bio13, bio15 and bio16 (i.e., precipitation of wettest month, precipitation seasonality, precipitation of wettest quarter). The T allele of ch2_130 is private to the exotic landraces group (Supplementary Table S8). On chromosome 3, we found two more loci with an allele private to exotic landraces. Both of these MTAs were associated with isothermality and were detected within *Solyc03g117760* and *Solyc03g121660*, genes that encode an un-known protein and a homolog of a zinc-finger protein, respectively (Supplementary Table S9).

We identified three additional MTAs associated with precipitation on chromosome 3. The loci ch3_275 and ch3_276 were correlated to bio 13 (i.e., precipitation of wettest month) and were both identified within *Solyc03g121000*, a gene encoding a PAF1-like protein (Supplementary Table S9). This protein regulates all stages of the RNA polymerase (Pol) II transcription cycle and in *Arabidopsis* it has been shown to transcriptionally regulate FLOWERING LOCUS C [73,74].

On chromosome 5, we detected a high number of MTAs. One within *Solyc05g015510* that it is associated with precipitation (i.e., precipitation of driest month) and encodes SlySBP10 a SQUAMOSA promoter-binding protein (Supplementary Table S9). This protein family has key roles in plant growth and development, including flowering time, shoot architecture and fruit setting and ripening [75,76,77,78]. We singled out other MTAs along the remaining chromosomes in regions that might be involved in responses to different climatic factors. Examples include *Solyc06g083150*, located 127 kb from a FRIGIDA-like gene. In *Arabidopsis*, FRIGIDA regulates flowering transition (vernalization) by activating a central flowering repressor that is encoded by flowering locus C [79]. Others are *Solyc07g055660*, located 26 kb from a heat-stress transcription factor; *Solyc08g076930*, a transcription factor for jasmonic acid 3 that is 72 kb from a RAMOSA transcription factor (Supplementary Table S9). In maize, RAMOSA genes act during early inflorescence development and determine the fate of axillary meristems [80].

## 4. Discussion

We have here investigated the value of a wide collection of tomato landraces for association mapping and detection of both novel loci and candidate genes that might be useful for designing future genomic-based breeding strategies or tomato gene-editing approaches. The collection is enriched by Italian, in particular Sardinian, landraces that represent a wealth of genetic local resources that might be exploited in future conservation and breeding studies. We investigated the collection in different environments and for different traits, including bio-climatic variables recorded for the country of cultivation. We also tested three different GWA methods to determine whether relevant differences might emerge from conventional and digital phenotyping.

### 4.1. Phenotypic and Molecular Diversity

The diversity levels and heritabilities measured across phenotypic traits showed that our tomato collection is adequate for GWAS. We have, indeed, shown that these materials are widely diverse for all of the investigated traits. Sardinian landraces in particular show a level of diversity similar to that of the group of exotic landraces. This is particularly relevant if we consider that the exotic landraces were chosen from among those available at the Centre for Genetic Resources (CGN, NL) to be a worldwide sample. On the other hand, the higher levels of genetic diversity observed within modern and vintage cultivars underlines that the breeding programs might have led to inclusion of different loci in these accessions [11,16].

Previous studies performed on tomato landraces and commercial cultivars using SNP markers revealed comparable levels of diversity and similar levels of heterozigosity when looking at groups of landraces from different regions [16,81,82,83]. Moreover, the partitioning of genetic variance has indicated that most of this diversity resides within varietal groups and not between them. Interestingly, novel alleles detected within both the Sardinian and the exotic landraces indicated that we have assembled a wide heterogeneous group with some unique diversity, which demonstrates the value of incorporating local and regional accessions when attempting to enrich the reservoir of crop genetic diversity. Indeed, nine of the 52 private alleles from the exotic landraces were associated with climate variables (mainly precipitation related). Private alleles are important because they indicate the presence of loci that can be exploited in future breeding and conservation programs or in genetic diversity studies [84]. For us, this is particularly interesting if we consider that these loci that are also associated to climatic variables, might be under selection. On the other hand, none of the 11 Sardinian private alleles were associated to any trait in this study.

The genetic distance observed among the groups of varieties was not high, which is consistent with the nature of domesticated tomato, for which high phenotypic but low genetic variation has been shown previously [1,11,16,85]. Concurrently, when we used all of the traits to investigate the phenotypic variation among accessions, the three main phenotypic clusters did not show any straightforward overlap with the genetic subdivision. This has been previously observed in studies on tomato and pepper [11,86]. Nonetheless, we observed a significant correlation between genetic and phenotypic distances and a clear tendency of accessions to group according to a small number of key traits, including fruit size and shape, pericarp thickness, carotenoid content and sugar content, similar to previous studies [11,16,81]. On the other hand, we did not observe any clear correlation between the genetic or phenotypic subdivisions and the provenience of our materials, as the landraces (either exotic or Sardinian) and the modern/vintage cultivars were distributed all over the detected groups.

### 4.2. Genome-Wide Association Study Results

The population structure and the linkage disequilibrium analyses performed prior to GWAS have further shown that our population was adequate for association analysis. While the LD levels and LD decay on some chromosomes were comparable to those from previous studies, these were quite variable among chromosomes [16,82,87]. In particular, the high levels of LD observed on chromosome 5 were similar to those from Ruggieri et al. [88], who used a set of genomic SNP markers to characterise a collection of cultivated tomatoes. To account for the high LD levels and decay on some chromosomes, following the example of previous studies [89,90], we reduced the average LD within chromosomes by correcting for population structure and genotype relatedness.

Among the methods used to perform these GWAS, FarmCPU and QTCAT shared the highest numbers of associations while the standard MLM method, showed the least number of shared associations with the other two methods. These data indicate that some associations are highly robust and detected by all methods and that weaker associations might best be identified using multiple strategies. In particular we also chose to use QTCAT to determine whether the associations detected were comparable with those from previous consolidated GWAS models [17,19,20,21]. Few studies have exploited the QTCAT model until now and results have shown consistent results with previous methods [22,91]. The present results indeed show that this method is efficient and has allowed the identification of novel associations that MLM and FarmCPU did not detect. In particular, the MLM model showed the poorest results.

Differently from many GWA studies that rely on the genomic variation, the present study was based on the transcriptome variation among genes and accessions. The RNAseq based SNPs genotyping has been proven to be valuable for QTL mapping and functional and evolutionary studies [92,93]. Concurrently, in the present study, the variation among landraces and cultivars at the RNAseq level was sufficient to detect numerous associations. Indeed, we showed the association between diverse crop traits to previously identified candidate genes and we were able to suggest that some of the target genes might either have pleiotropic effects on different traits or show co-expression due to linkage. This has also been observed in previous GWAS and QTL studies [14,15,16,94]. As our population was variegated with no clear breeding history, such as in Blanca et al. [81] and Lin et al. [2], the observed genetic diversity might reflect complex metapopulation dynamics, with some loci under divergent selection and others under balancing selection, as previously seen in tomato, sorghum and maize [95,96,97,98]. Indeed, the level and structure of the diversity of cultivated tomato derives from historical and recent gene flow between wild tomato species and cultivars that allowed the dispersal of local varieties across a wide range of environments, with a complex evolution (i.e., domestication, improvements) [2,97,99].

The GWAS results have mainly targeted genomic regions that underlie genes relevant for fruit size and shape traits. This is a direct consequence of our phenotypic analysis, which mainly focused on fruit traits, in line with the relatively thorough phenotypic characterization, as either detected by conventional or digital phenotyping. This is also a good way to evaluate the appropriateness of the present collection for GWAS studies. Indeed, a large number of associations were detected with data from the Tomato Analyzer, which was also successfully used in previous studies [58,86,100]. In particular, this method has been effective in associating different fruit shape categories to different alleles of SUN, OVATE, FASCIATED and LOCULE NUMBER, genes that control the elongated and flattened fruit shape in tomato [55,58,101,102].

Additional traits identified by these authors as predictive for these categories were fruit shape index, distal end protrusion, width widest position, proximal end blockiness, rectangular, distal angle and proximal eccentricity. Here, we also detected many of these associations, such as that with Proximal_angle_macro near OVATE or with fruit-shape-related traits near FASCIATED. However, interestingly, along with these associations, we also detected further associations linked to SlCLV3 (homolog of *Arabidopsis* CLAVATA3), which underlies the *fasciated* mutant phenotype [56,101,103,104,105], and a TONNEAU1-recruiting-motif gene. Together with OVATE, different genes from this family regulate early ovary development, which affects final fruit shape [53].

Along with studies mainly based on mutants, more recent GWAS based on genomic SNP, have detected significant polymorphisms within the same region [16,106]. Among associations detected for the fruit traits described in the present study, some were exclusive to conventional phenotyping and others to digital phenotyping. This demonstrates that the ability of the human eye to synthesize multiple features cannot yet be completely replaced by the available technology.

Many MTAs located either within or near transcription factor genes influenced fruit setting (OVATE-like, WUSCHEL-related, YABBY-like). These show that this combined regional and global tomato collection is useful for targeting genes with prominent roles in fruit. As an example, a single SNP (ch2_151) was identified within a gene that encodes a proteasome subunit α type protein that appears to have a key role during fruit setting [107,108]. This MTA was identified by all three of the GWAS methods for multiple fruit traits (detected by both conventional and precision phenotyping) near ANANTHA, which regulates inflorescence branching and floral organ identity [109], and in the region of additional MTAs for fruit shape and size.

Together, these results suggest that the diversity of this germplasm is sufficient to gain further insights into the role and function of genes that underlie important traits. Indeed, further associations were found in regions that were previously targeted as important for fruit color, carotenoids and sugar content, while others targeted novel genes that might be of relevance for quality traits, such as fruit color and β-carotene content on chromosomes 1 and 4 [59,110,111,112].

Using GWAS on SNPs from RNA-Seq extracted from mature fruit, we did not expect a direct implication for most of the present polymorphisms in the expressions of genes involved in plant development and phenology. However, we would expect a relevant number of these genes to be in LD (physical or functional) with others expressed in the fruit tissue. The significant RNA variation has indeed allowed the identification of associations for growth habit. These were often at short distances from transcription factors that have determinant roles in the regulation of plant growth and development, including tomato ripening and responses to environmental stimuli [64,113,114,115].

As revealed by a recent study from Wu and colleagues [53], genes of the OVATE and TONNEAU1 recruiting motif families not only control fruit shape in different domesticated plants (e.g., tomato, potato, melon, cucumber) but also interact to regulate other plant organs, such as leaves and flowers. This is in line with the present results, as among the different MTAs, some show joint association (either functional or as a result of hitchhiking during selection) with traits that underlie fruit development and plant and flower development, as also flowering. The MTA within *Solyc06g066320*, the gene encoding for a IWS1 transcription factor, is particularly interesting because this protein is highly conserved in different organisms (from humans to yeast) in *Arabidopsis* it is required for gene expression induced by brassinosteroids [60,116]. Moreover, the region around this *gene* spanning nearly 0.5 Mb, is enriched of genes encoding for different transcription factors, also related to fruit development and ripening [114,117]. In particular, the closest to IWS1 is a homolog to KANADI2, that together with KANADI1, appeared to be involved in the development of the carpel in *Arabidopsis*, as well as in the establishment of polarity of lateral organs, including leaf and flower, in *Arabidopisis*, rice and other species [118,119,120]. The function of *Solyc06g066320*, together with its simultaneous associations with different traits (i.e., fruit quality, inflorescence, fruit shape and size), suggest a possible pleiotropic role for this gene. Being pleiotropy a target for many GWA studies, variable strategies have been designed for the identification of causal variants and their roles that will help to extract the huge amount of information that is being produced [13,121,122].

### 4.3. Climatic Data

Previous studies based on climatic data have revealed gene expressed regions that might be involved in the answer to different environments [123,124,125,126,127,128]. They also suggest a role for the target genes in responses to environmental effects during plant development, such as those involved in regulation of the circadian clock, DNA methylation or flowering delay.

Among the most interesting results, we found MTAs identified on chromosome 2 for variables related to precipitation, such as ch2_130, with its “T” allele being private to the exotic landrace population. This MTA is located within *Solyc02g071510*, which encodes a transcription factor (GTE12) that in *Arabidopsis* is implicated in the cytosolic Ca^2+^ increase after application of stress, such as heat, drought and cold [129,130]. Three additional MTAs were also private alleles of the exotic landrace population and all were associated to bioclimatic variables, which suggest that these variants might be a signal of adaptation. Previous studies, performed on sorghum landraces have evidenced that the environment of origin have had a significant role in shaping SNP allele variation and suggested that these genomic signatures of adaptation might be used for crop improvement [126,127,128,131].

Further associations were detected across the remaining chromosomes in genomic regions that appear to be involved in plant responses to climatic changes. As an example, we indicate the MTAs located within a gene that encodes a heat stress transcription factor or within SlMYC2, a jasmonic acid transcription factor involved in fruit chilling tolerance in tomato [132]. Others are located near genes that encode a FRIGIDA-like or other heat-stress proteins, as well as in close proximity to genes implicated in the regulation of flowering or inflorescence architecture. Since tomato cultivation is hindered by high temperature and drought, these results might help to target genes that will be useful to develop new varieties that are better adapted to changing environments.

The present results indicate that we can select the accessions that carry the polymorphic alleles to create specific crosses with controlled fruit size and shape and, for example, improved quality characteristics or that might be linked to specific climate responses.

## 5. Conclusions

We demonstrate here the value of exploring the genetic diversity of local and regional accessions, to exploit the variation of this genetic diversity reservoir to gain knowledge and facilitating crop improvements. Our results indicate that, as well as the relevant associations detected for fruit size, shape and quality, there is correlation between the expression of fruit traits and other traits related to plant development and flowering, and the likely response to climate variations. As recently demonstrated in tomato, the human-driven selection for specific traits during domestication has resulted in direct effects on other secondary traits or indirect influences in the expression of other genes to linkage drag [3,133]. Some of the present outcomes might be used to investigate gene functions in basic genetic studies or in the post-GWAS era, to be exploited in gene editing programs, and for genomic selection and breeding. The present collection is enriched in landraces that, along with the wild species, represent a reservoir of diversity that might translate into improved crops and/or crops adapted to ever-changing consumer preferences.

## Figures and Tables

**Table 1:** ANOVA analysis performed across 122 tomato accessions for 14 conventional quantitative traits evaluated in 2012 and 2013. Year (*Y*), genotype (*G*) and genotype × year (*G × Y*) interactions were considered as effects of the model.

Trait	Year				Genotype				Genotype × Year					h^2^_B_	
	DF	SS	F		DF	SS	F		DF	SS	F		2012	2013	2012/2013
DTFs	1	508,837.7	26,547.5	****	121	43,086.7	18.6	****	121	15,408.1	6.6	****	51.2	55.0	34.4
DTFt	1	125,556.0	6876.4	****	121	41,182.4	18.6	****	121	14,045.8	6.4	****	51.2	58.0	35.9
FRI	1	247,271.0	7000.0	****	121	31,918.6	7.5	****	121	22,707.8	5.3	****	30.7	43.4	11.8
NFI	1	170.8	8.3	**	121	25,164.3	10.1	****	121	8662.5	3.5	****	65.7	57.6	36.3
LLE	1	577.3	36.5	****	121	12,356.6	6.5	****	121	3732.4	1.9	****	42.5	25.9	28.6
LWI	1	2.2	0.1	n.s.	121	19,444.8	6.8	****	121	6552.4	2.3	****	43.9	31.7	27.3
LL/W	1	0.6	29.7	****	121	10.0	4.1	****	121	3.9	1.6	****	26.0	42.1	20.0
FWG	1	403.6	0.1	n.s.	121	13,339,038.6	33.2	****	121	1,192,320.9	3.0	****	67.8	62.2	59.9
FLE	1	27.2	55.9	****	121	4260.4	72.4	****	121	217.2	3.7	****	78.2	80.9	74.2
FWI	1	11.5	12.1	***	121	8233.2	71.4	****	121	344.9	3.0	****	78.9	80.6	76.6
FL/W	1	1.8	126.9	****	121	209.2	120.4	****	121	4.4	2.5	****	85.1	89.3	85.8
NOL	1	56.3	16.1	****	121	30,730.5	72.5	****	121	1009.8	2.4	****	81.2	80.4	79.0
PTK	1	2.6	347.0	****	121	26.7	29.1	****	121	2.5	2.7	****	55.8	70.5	58.8
BRIX	1	239.9	610.7	****	121	798.1	16.8	****	121	128.8	2.7	****	44.5	55.5	42.3

DF, degrees of freedom; SS, sum of squares; F, F ratio; h^2^_B_, broad sense heritability; n.s., not significant; *, *p* < 0.05; **, *p* < 0.01; ***, *p* < 0.001; ****, *p* < 0.0001. DTFs, days to flowering from sowing (days); DTFt, days to flowering from transplanting (days); FRI, flowering-ripening interval (days); NFI, number of flowers per inflorescence; LLE, leaf length (cm); LWI, leaf width (cm); LL/W, leaf length/width; FWG, fruit weight (g); FLE, fruit length (cm); FWI, fruit width (cm); FL/W, fruit length/width; NOL, number of locules; PTK, pericarp thickness (cm); BRIX, degrees Brix (°Brix).

**Table 2:** Genetic diversity indices calculated within the different accessions groups and based on the 2470 single nucleotide polymorphisms (SNPs).

Group	Sample Size	Na	Ne	No. PA	He	uHe
ELR	48	1.94	1.23	50	0.16	0.16
SLR	61	1.88	1.23	11	0.16	0.16
CV	11	1.92	1.44	1	0.28	0.29
Overall	120	2.00	1.25		0.18	0.18

Na, No. of different alleles; Ne, No. of effective alleles; I, Shannon’s information index; No. PA, No. of alleles unique to a single population; He, expected heterozygosity; uHe, unbiased expected heterozygosity. ELR, exotic landraces; SLR, Sardinian landraces; CV, modern and vintage cultivars.

**Table 3:** F_ST_ values (below diagonal) and relative significance of the test (above diagonal) and proportion of shared alleles between pairs of sub-populations based on the 2470 SNPs.

Comparison among Groups	ELR	SLR	CV
Genetic differentiation (F_ST_)			
ELR		***	**
SLR	0.04		*
CV	0.09	0.08	
Proportion of shared alleles			
ELR			
SLR	0.93		
CV	0.87	0.88	

ELR, exotic landraces; SLR, Sardinian landraces; CV, modern and vintage cultivars; n.s., not significant; *, *p* < 0.05; **, *p* < 0.01; ***, *p* < 0.001.

**Table 4:** Linkage disequilibrium levels overall and within chromosomes, as calculated using standard r^2^ and (r^2^) corrected for population structure (r_s_^2^), kinship (r_v_^2^) and both population structure and kinship (r_vs_^2^).

Chromosome	Mean r^2^	r^2^ Decay at 0.30 Mb	Mean r_s_^2^	r_s_^2^ Decay at 0.22 Mb	Mean r_v_^2^	r_v_^2^ Decay at 0.14 Mb	r_vs_^2^	r_vs_^2^ Decay at 0.14 Mb
chr1	0.06	0.19	0.05	0.34	0.05	0.37	0.05	0.37
chr2	0.14	0.63	0.11	0.91	0.11	1.02	0.11	0.94
chr3	0.08	0.21	0.07	0.32	0.07	0.34	0.07	0.33
chr4	0.13	0.70	0.11	1.13	0.11	0.80	0.11	0.81
chr5	0.34	42.57	0.17	8.74	0.17	15.97	0.17	8.12
chr6	0.09	0.35	0.09	0.69	0.09	0.83	0.09	0.84
chr7	0.15	0.75	0.13	1.29	0.13	1.65	0.13	1.65
chr8	0.06	0.12	0.05	0.21	0.05	0.27	0.05	0.27
chr9	0.13	0.40	0.12	0.68	0.12	1.03	0.12	1.04
chr10	0.08	0.09	0.07	0.17	0.07	0.25	0.07	0.25
chr11	0.13	0.74	0.09	0.52	0.09	0.55	0.09	0.55
chr12	0.24	2.11	0.23	3.63	0.23	1.36	0.23	1.37
Mean	0.15	0.63	0.11	0.81	0.07	0.80	0.07	0.80

The mean linkage disequilibrium (LD) levels over all chromosomes were r^2^ = 0.15, r_s_^2^ = 0.11 and r_vs_^2^ = 0.07, whereas the estimates of the unlinked pairwise loci (among chromosomes) indicated r^2^ = 0.19, r_s_^2^ = 0.12 and r_vs_^2^ = 0.09 as the threshold above which loci can be assumed to be in LD. There were fewer SNPs across centromeres and the highest LD levels were usually observed around peri-centromeric areas.

**Table 5:** Distribution and number of marker-trait associations detected along tomato chromosomes. Data are presented according to the GWAS method and then trait type. FRUIT_SHAPE_CP and FRUIT_SIZE_CP/DP, fruit shape and size, respectively, based on conventional/digital phenotyping.

Method/Trait Type	CHR1	CHR2	CHR3	CHR4	CHR5	CHR6	CHR7	CHR8	CHR9	CHR10	CHR11	CHR12	TOTAL
FarmCPU	30	30	36	26	22	17	8	17	9	19	44	7	265
CLIMATE	2	5	13	4	6	4	3	2	2	6	9	3	59
FRUIT_QUALITY	4	1	2	1	1				1	1	1		12
FRUIT_SHAPE_CP	3	5	2	2	4	1		3	2	2	4		28
FRUIT_SHAPE_DP	6	6	8	13	6	2	1	9	3	2	15	3	74
FRUIT_SIZE_CP	2	3	4		1	2	1			2	3		18
FRUIT_SIZE_DP	8	5	2	1	3	6		2		2	6		35
GROWTH	1					1				1	2		5
INFLORESCENCE	2	1	1	1		1				2	3	1	12
LEAF TRAITS	1	2	3	3	1		3	1		1	1		16
PHENOLOGY	1	2	1	1					1				6
GAPIT.MLM	5	4	3	6	5	2		6			8	1	40
CLIMATE	2		3	2	5			2				1	15
FRUIT_QUALITY	3					1							4
FRUIT_SHAPE_CP		3		1				1			4		9
FRUIT_SHAPE_DP		1		3		1		3					8
FRUIT_SIZE_CP											4		4
QTCAT	29	33	45	5	12	14		10	7	13	57	6	231
FRUIT_QUALITY		1	1			1			2		1		6
FRUIT_SHAPE_CP	1	4	6					2		3	7	1	24
FRUIT_SHAPE_DP	2	14	19	5	6	8		5	3	3	13	2	80
FRUIT_SIZE_CP	7	2	6		1					3	4	3	26
FRUIT_SIZE_DP	15	12	13		5	4		3	2	4	24		82
INFLORESCENCE	1					1					8		10
LEAF TRAITS	1												1
PHENOLOGY	2												2
TOTAL	64	67	84	37	39	33	8	33	16	32	109	14	536

## Data Availability

Raw RNA-Seq reads have been deposited into the NCBI sequence read archive (SRA) under accession PRJNA646818. All data necessary for confirming the conclusions of the article are present within the article, Figures, and Tables, and within Supplementary Tables and Figures.

## Supplementary Materials

The following are available online at https://www.mdpi.com/2073-4425/11/11/1387/s1, Figure S1: Tomato fruit collected for the carotenoids content analysis. Different varieties of the tomato collection harvested at the breaker (greenish color) and ripe (red color) stages (Photograph: C.M. Posadinu). Figure S2: Estimates of Pearson’s correlations among all of the traits recorded. The red (negative) to blue (positive) color palette is used to indicate the strength of the correlation. White indicates no correlation. The codes for each trait are specified in Table S2. Figure S3: Most likely number of populations as obtained from STRUCTURE (A) and DAPC (B) analyses. (A) Delta K for increasing K performed over the 120 tomato accessions characterized by RNA-Seq analysis. Ten runs were performed for each K value. (B) The most likely number of genetic groups of four, as indicated by the lower Bayesian information criterion (BIC) value. Figure S4: Population structure as obtained from morphophenotypical and quality traits (A) and genetic analysis (B). A certain level of overlap is seen between the two clustering methods, although the overall variation captured by one of the two analyses is not fully representative of the other. Figure S5: Linkage disequilibrium (LD) versus physical distance, from chromosome 1 to chromosome 6. Left to right: Each plot represents four different LD measures per single chromosome, for the usual r^2^ value, the r_s_^2^ measure (r^2^ corrected by the population structure), the r_v_^2^ (r^2^ corrected by the kinship), the r_vs_^2^ measure (r^2^ corrected by both the kinship of individuals and the population structure). Figure S6: Linkage disequilibrium (LD) *versus* physical distance, from chromosome 7 to chromosome 12. Left to right: Each plot represents four different LD measures per single chromosome, for the usual r^2^ value, the r_s_^2^ measure (r^2^ corrected by the population structure), the r_v_^2^ (r^2^ corrected by the kinship), the r_vs_^2^ measure (r^2^ corrected by both the kinship of individuals and the population structure). Table S1: Accessions, names, origin, donors and details for the analysis performed on each accession. Table S2: Phenotypic traits (conventional, and from Tomato Analyzer) and carotenoid contents as registered for the tomato collection. Bio-climatic data are also given (as obtained for the 108 geo-referenced accessions). Table S3: Significant differences among the tomato accessions for the conventional quantitative traits in 2012 and 2013. Table S4: Shannon–Weaver index for the qualitative traits evaluated in 2012 and 2013. Table S5: Distribution and variance of the digital phenotyping descriptors, as evaluated in 116 tomato accessions in 2013. The ANOVA was applied to the data to test for significant differences among traits. Table S6: Significant differences among genotypes of cultivated tomato for all of the compounds analyzed. Table S7: Loading of the PCA—see Figure 1 for PC plot—performed using phenotypic traits, carotenoids and bioclimate variables. Table S8: Summary of private alleles by population. When the allele is also associated to a specific trait—based on GWAS results—it is indicated in column E. Table S9: Gene ontology based on Genome V2.4 and V2.5 and Tomato Genome_4.0. The columns from S to AB indicate the SNPs that have been detected as associated to different traits. Additional notes are given for each marker-trait association when present. Table S10: Distribution of marker-trait associations on the different chromosomes and genes within each chromosomes. Data are subdivided into groups of different phenotypic traits. Files (.txt format) with all the data are also given: Files with SNP and morphologic/quality/climatic data: SNP dataset in hapmap format: tmt_120g_2470SNPs.hmp.txt; Carotenoid data: tmt_carotenoids_traits_BLUPs.txt; Climatic data: tmt_climate_traits.txt; Conventional morphologic traits: tmt_conventional_traits_BLUPs.txt Morphologic traits as obtained from digital phenotyping: tmt_TomatoAnalyzer_traits_BLUPs.txt; Output of population structure: tmt_pop_str_K3.txt; Kinship matrix: kinship_120i_2470SNPs.txt.
