# Supplementary material for: GWAS Based on RNA-Seq SNPs and High-Throughput Phenotyping Combined with Climatic Data Highlights the Reservoir of Valuable Genetic Diversity in Regional Tomato Landraces
Source: Genes (Basel). 2020 Nov 23;11(11):1387. doi: 10.3390/genes11111387 (PMC7709041; doi:10.3390/genes11111387)
Supplement: Supplementary file 1 [file genes-11-01387-s001.zip › Supplementary_Data_Tables_Figures/Supplementary_Figures.docx]

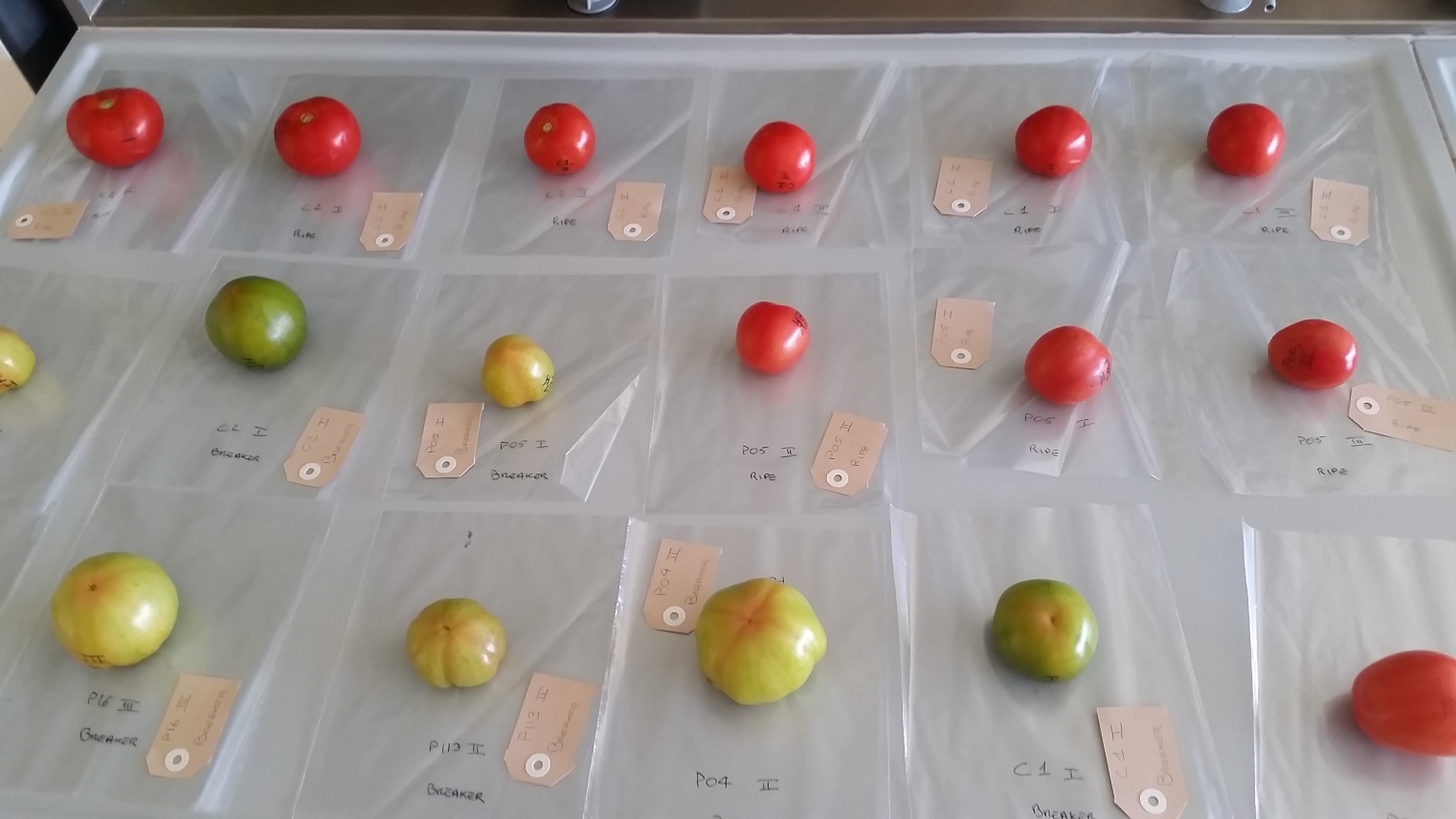


**Figure S1. Tomato fruit collected for the carotenoids content analysis.** Different varieties of the tomato collection harvested at the breaker (greenish color) and ripe (red color) stages (Photograph: C.M. Posadinu).


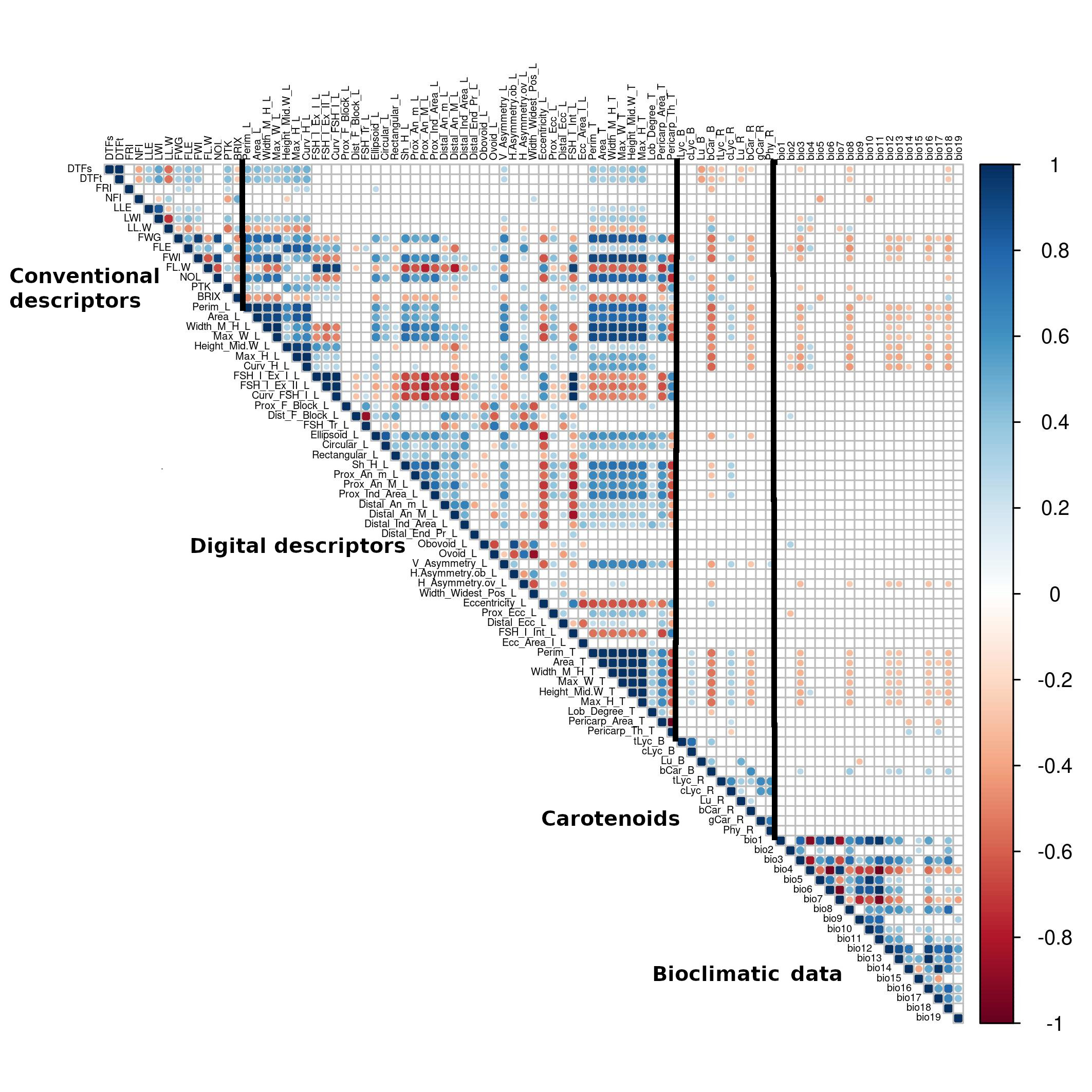


**Figure S2. Estimates of Pearson’s correlations among all of the traits recorded.** The red (negative) to blue (positive) color palette is used to indicate the strength of the correlation. White indicates no correlation. The codes for each trait are specified in Table S2.


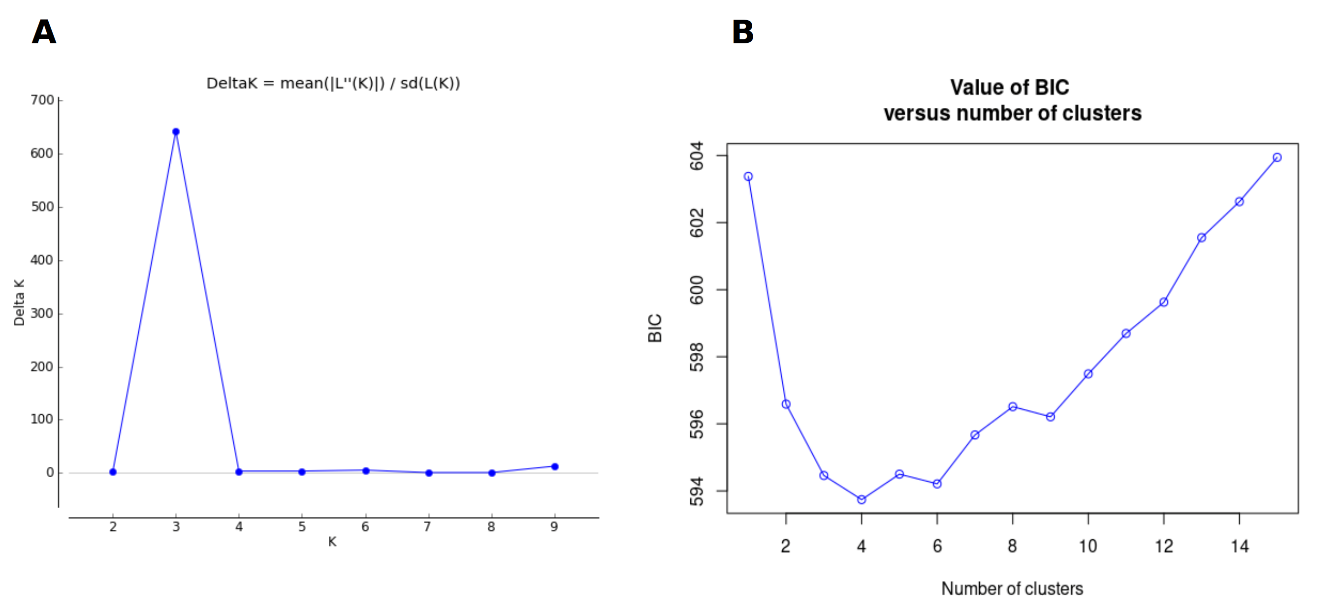


**Figure S3. Most likely number of p****opulations as obtained from STRUCTURE (A) and DAPC (B) analyses. A)** Delta K for increasing K performed over the 120 tomato accessions characterized by RNA-Seq analysis. Ten runs were performed for each K value. B) The most likely number of genetic groups of four, as indicated by the lower Bayesian information criterion (BIC) value.

**
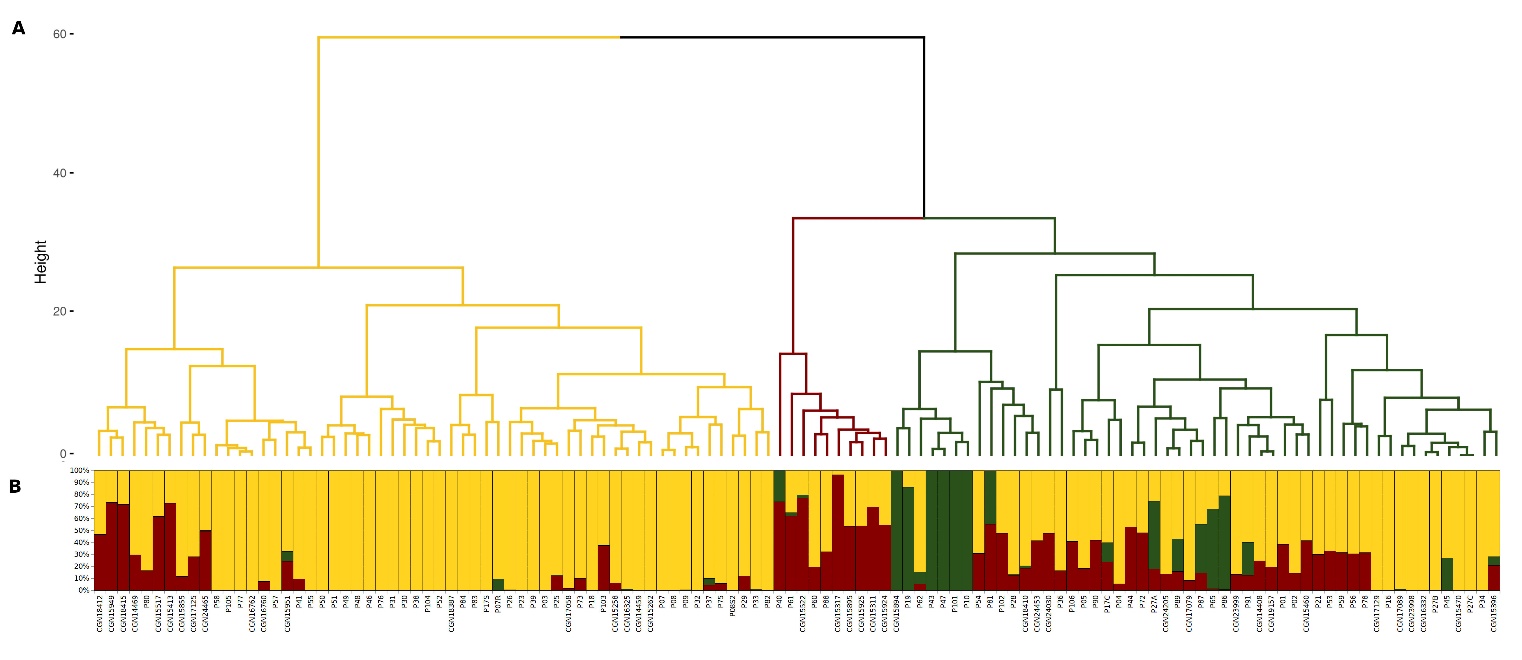
**

**Figure S4. Population structure as obtained from morphophenotypical and quality traits (A) and genetic analysis (B).** A certain level of overlap is seen between the two clustering methods, although the overall variation captured by one of the two analyses is not fully representative of the other.


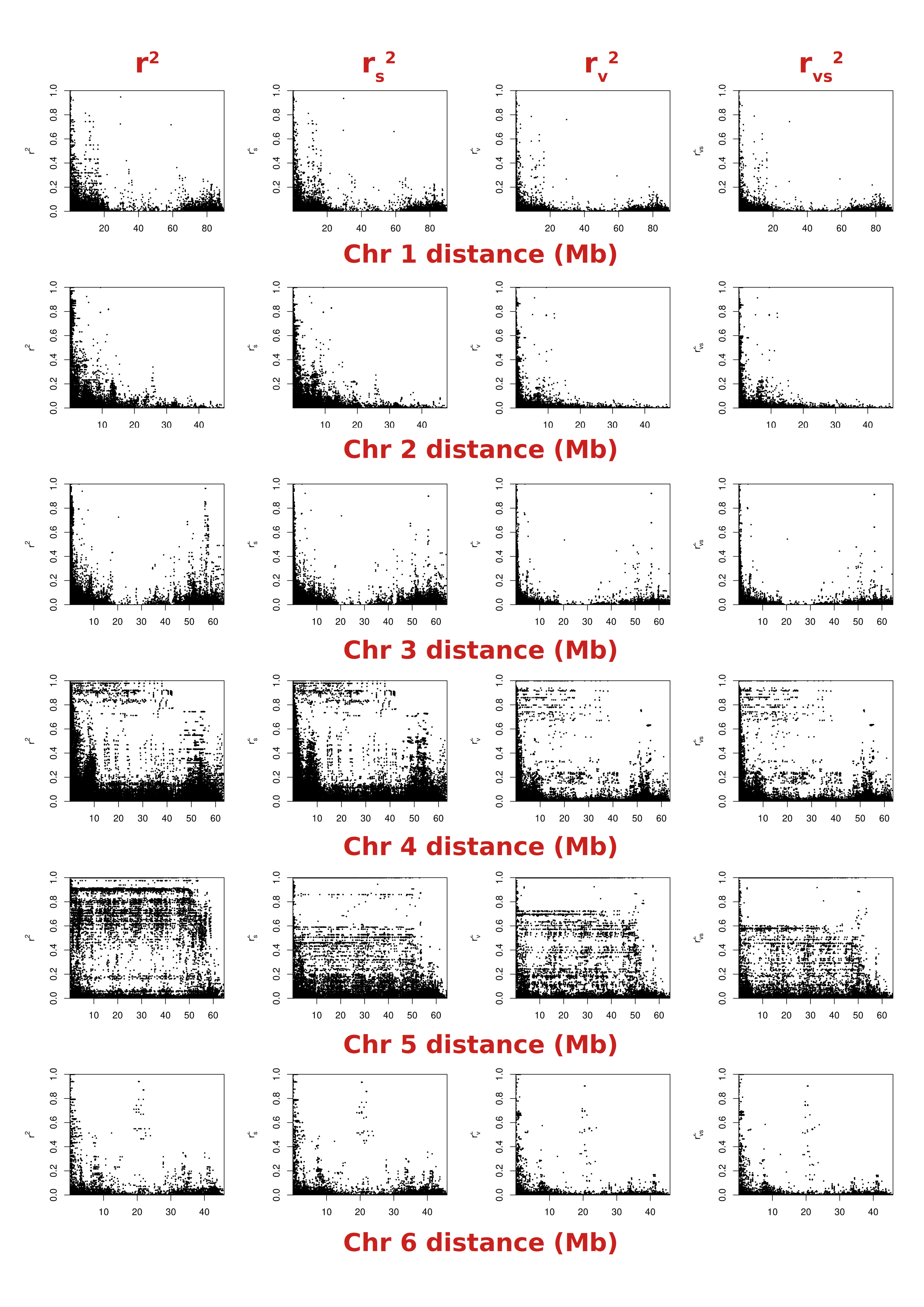
**Figure S5. Linkage disequilibrium (LD) *versus* physical distance, from chromosome 1 to chromosome 6.** Left to right: Each plot represents four different LD measures per single chromosome, for the usual r^2^ value, the r_s_^2^ measure (r^2^ corrected by the population structure), the r_v_^2^ (r^2^ corrected by the kinship), the r_vs_^2^ measure (r^2^ corrected by both the kinship of individuals and the population structure).


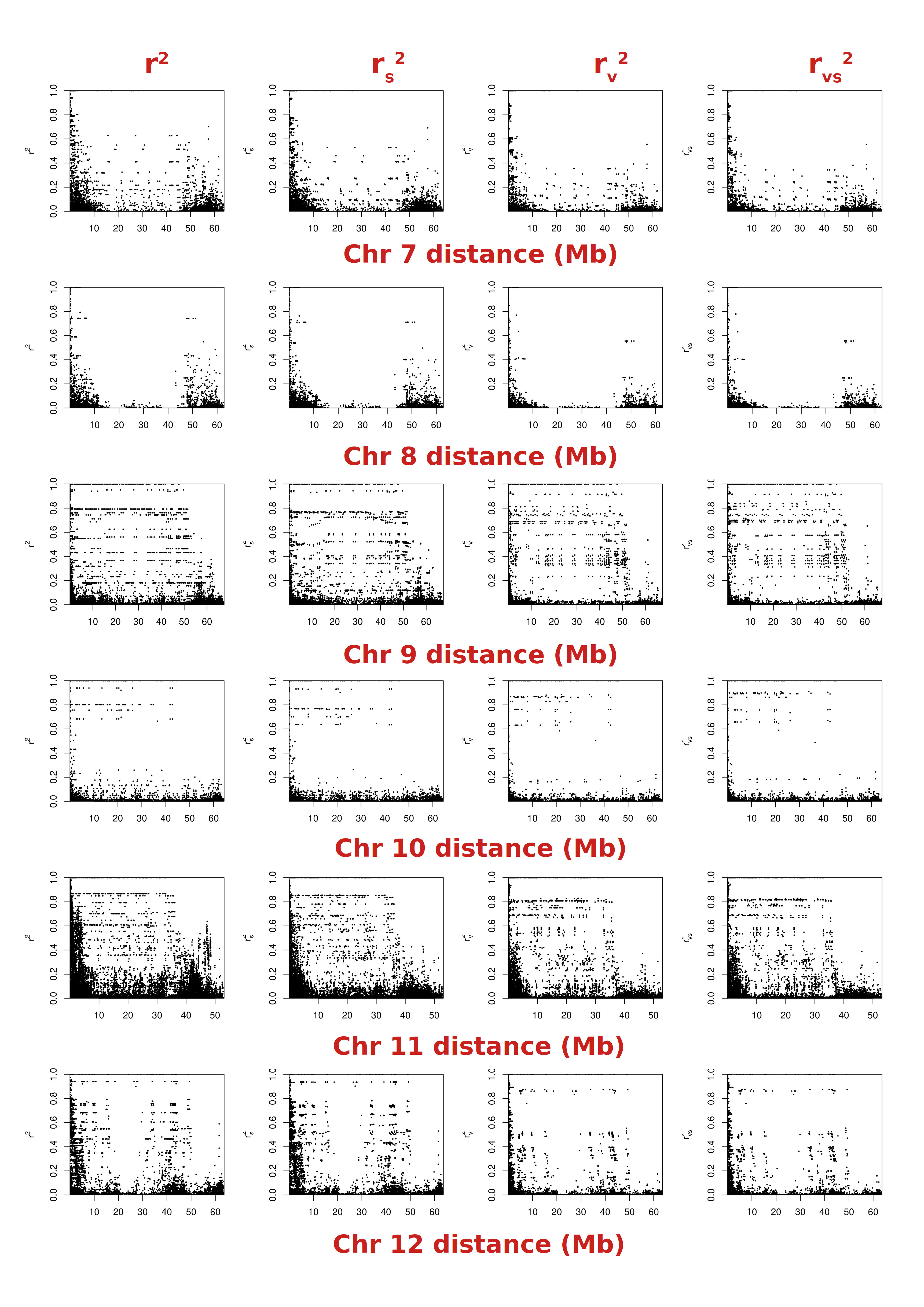


**Figure S6. Linkage disequilibrium (LD) *versus* physical distance, from chromosome 7 to chromosome 12.** Left to right: Each plot represents four different LD measures per single chromosome, for the usual r^2^ value, the r_s_^2^ measure (r^2^ corrected by the population structure), the r_v_^2^ (r^2^ corrected by the kinship), the r_vs_^2^ measure (r^2^ corrected by both the kinship of individuals and the population structure).
